# Adopting a multidisciplinary telemedicine intervention for fall prevention in Parkinson’s disease. Protocol for a longitudinal, randomized clinical trial

**DOI:** 10.1371/journal.pone.0260889

**Published:** 2021-12-21

**Authors:** Esther Cubo, Alvaro Garcia-Bustillo, Alvar Arnaiz-Gonzalez, Jose Miguel Ramirez-Sanz, Jose Luis Garrido-Labrador, Florita Valiñas, Marta Allende, Jeronimo Javier Gonzalez-Bernal, Josefa Gonzalez-Santos, José Francisco Diez-Pastor, Maha Jahouh, Jana Arribas, Jose Trejo

**Affiliations:** 1 Hospital Universitario Burgos, Burgos, Spain; 2 Facultad Ciencias de la Salud, University of Burgos, Burgos, Spain; 3 Facultad Ingeniera Informática, Universidad de Burgos, Burgos, Spain; Universidade Federal do Rio Grande do Sul, BRAZIL

## Abstract

**Background:**

Approximately 40–70% of people with Parkinson’s disease (PD) fall each year, causing decreased activity levels and quality of life. Current fall-prevention strategies include the use of pharmacological and non-pharmacological therapies. To increase the accessibility of this vulnerable population, we developed a multidisciplinary telemedicine program using an Information and Communication Technology (ICT) platform. We hypothesized that the risk for falling in PD would decrease among participants receiving a multidisciplinary telemedicine intervention program added to standard office-based neurological care.

**Objective:**

To determine the feasibility and cost-effectiveness of a multidisciplinary telemedicine intervention to decrease the incidence of falls in patients with PD.

**Methods:**

Ongoing, longitudinal, randomized, single-blinded, case-control, clinical trial. We will include 76 non-demented patients with idiopathic PD with a high risk of falling and limited access to multidisciplinary care. The intervention group (n = 38) will receive multidisciplinary remote care in addition to standard medical care, and the control group (n = 38) standard medical care only. Nutrition, sarcopenia and frailty status, motor, non-motor symptoms, health-related quality of life, caregiver burden, falls, balance and gait disturbances, direct and non-medical costs will be assessed using validated rating scales.

**Results:**

This study will provide a cost-effectiveness assessment of multidisciplinary telemedicine intervention for fall reduction in PD, in addition to standard neurological medical care.

**Conclusion:**

In this challenging initiative, we will determine whether a multidisciplinary telemedicine intervention program can reduce falls, as an alternative intervention option for PD patients with restricted access to multidisciplinary care.

**Trial registration:**

**ClinicalTrials.gov Identifier**: NCT04694443.

## Background

The Parkinson’s disease (PD) population is mainly sedentary, causing a dramatic reduction of physical activity compared to healthy older adults, even early in the disease [1, 2]. As PD progresses, falls are often harmful and very common [3]. Approximately 40–70% of people with PD fall each year, and one-third fall repeatedly [3, 4]. twice as likely as the healthy elderly population [5]. Evidence shows that falls significantly impact people’s lives with PD, including debilitating effects on confidence, decreased activity levels, and reduced quality of life [6–8]. Consequently, repeat falls are a risk factor that implies subsequent falls, often with such consequences as fractures, prolonged immobility and dependency, social isolation, and financially costly individuals and healthcare systems [9].

Although falling increases with disease severity, falls are even common in the early stages of the condition [10]. Anti-parkinsonian drugs are the main treatment to counter the symptoms that can cause PD patients to fall, while research into finding a cure continues. However, medication cannot adequately control reduced postural control and prevent falls, especially among patients with advanced PD [4]. Other fall-prevention strategies include educational support, physical exercise, and modification of environmental factors [3, 5, 8]. In this regard, behavioral change strategies provide a theoretically based approach for helping patients with PD develop the skills they need (e.g., goal setting, self-monitoring) for successful engagement in sustained exercising, and therefore fall prevention [11]. Randomized clinical trials published in the literature have concluded that exercise as a single intervention can prevent falls among community-dwelling patients with PD and can be particularly beneficial for maintaining postural control, mobility, and daily living activities [12]. Also, there is growing evidence that remote monitoring of physical activity is also feasible in neurological diseases, including PD [11, 13]. In Spain, PD associations often offer long-term physical non-pharmacological therapies for PD, including physical, occupational, speech therapies, and psychological support. Based on the estimated average prevalence rates of PD in Spain of 682.2/10^5^ (IC: 127.4/10^5^−1491.7/10^5^) [14], but only 14% of these patients receive non-pharmacological therapies provided by PD associations (Spanish PD Association. Unpublished data). This scenario has even worsened during the Covid-19 pandemic.

In this regard, the role of Information and Communication Technologies (ICT) in a home fall prevention program for patients living with PD could be crucial and a promising, cost-effective strategy, with the added advantage of reaching PD patients remotely [15, 16]. Different software solutions have been described on telerehabilitation, such as the Platform of Telemedicine (Teleriab), designed to offer telerehabilitation services. Health staff can contact patients through this web platform either over the phone or by using a videoconferencing option with one or more patients simultaneously or consecutively, respecting the patients’ privacy [17]. Other authors have provided telerehabilitation through videoconferencing via a smartphone or iPad with a (digitally simulated) avatar coach [18–20]. Previously, most published reviews have discussed only the short-term benefits of rehabilitation for people with PD. Studies designed to analyze the feasibility and cost-effectiveness of multidisciplinary telemedicine programs for fall prevention in PD are needed. Given the lack of scientific evidence, we hypothesized that a multidisciplinary telemedicine intervention program added to a standard in-office neurological care would be more effective than standard in-office neurological care to decrease the incidence of falls in PD. This study describes the methodology of a clinical trial designed to analyze the feasibility and cost-effectiveness of a low-cost telemedicine multidisciplinary telemedicine program to reduce the incidence of falls in addition to in-office usual care in a population with PD.

## Materials and methods

### Design

This study is a clinical trial protocol related to a future randomized controlled trial of an 8-month-follow-up study, multidisciplinary home-based telerehabilitation intervention, single-center, case-control, single-blinded, longitudinal, 8-month-follow-up study. This study is registered on clinicalTrials.gov Identifier, with the registration number NCT04694443. The protocol of this study followed the SPIRIT reporting criteria for protocol reports [21]. We will conduct this study at the Movement disorder unit of a tertiary hospital, Hospital Universitario Burgos, in collaboration with the Health Science and the Computer Engineering School at the University of Burgos, Spain.

### Specific goals

The first objectives of this study are to study and compare the intervention vs. the control group, in terms of 1) the reduction of fall incidence (total number of falls from baseline) with the implementation of an additional multidisciplinary telemedicine program; and 2) the cost-effectiveness of this telemedicine program compared to usual care. Secondary objectives are: 1) to study the feasibility and patients’ adherence to new technology (defined as patients retention > 80% during the study), 2) to study the clinical utility of using wearable sensors to monitor gait dysfunction and physical activity, 3) to analyze the duration of clinical benefits after finishing a telemedicine program (short-term vs. long-term benefits), and 5) to develop a full-stack using Big Data frameworks that facilitate communication between the patient and the occupational therapist using Artificial Intelligence (AI) techniques.

### Study population

We will invite non-demented, ambulatory patients aged > 18 years old, with established idiopathic PD based on the MDS-criteria [22, 23], with a medium-high risk of falling established by a history of at least one fall in the previous 12 months with a Hoehn & Yahr (HY) stage < 3 [24], a Montreal Cognitive Assessment (MoCA) score > 18 [25], without access to non-pharmacological therapies, and one of the two additional criteria including the presence of freezing of gait (FOG) or self-selected gait speed < 1.1 meters/second [26]. We will exclude patients with severe psychiatric or cerebrovascular diseases, severe traumatic head injuries, severe orthopedic lower limb or spine problems, peripheral neuropathy, rheumatological disease, or other systemic diseases or sensory deficiencies that, according to the investigator’s judgment, may interfere with the study.

### Ethical considerations

This study will be conducted according to the standards for Good Clinical Practice, the fundamental ethical principles established in the Declaration of Helsinki and the Oviedo Convention, and the requirements established in Spanish legislation in the research field. This project has been approved by the Comité Ético Complejo Universitario Burgos y Soria (Certificate number: CEIC 2112, October, 29^th^, 2019). All patients will read and sign the informed consent form before participating. The researchers will ensure that the anonymity of the participants is protected, and the data concerning people will remain confidential so that their identities and any identifying information are protected. The clinical records, research instruments, or any used documents that contain data from the participants will not be identified by the participant’s name but rather by a code, even when the information is submitted to regulatory institutions or sponsors. The researchers will protect the participants’ records, and the information on the codes, names, and addresses of the participants will remain confidential and available only to the researchers. Two copies of the consent forms will be provided, both of which will be signed by the participants, researchers, and guardians (or legal representatives). The researchers will store all files in a secure location in a single folder dedicated to this study. If a participant does not follow these rules, he or she will receive an original copy of the consent form. The results of this research may be presented at meetings or publications; however, the participants’ identities will not be revealed in these presentations.

The risks during the research are minimal or nonexistent, but the assessments and rehabilitation procedures may cause discomfort due to balance problems. If discomfort is experienced, the evaluations may be paused until medical interventions are performed, if necessary. However, if discomfort persists, the participant will be removed from the research, the data collected will be excluded from analysis, and the patient will not be reallocated to another group

### Quality assurance: Investigator team

The investigator team will be composed of two movement disorders MDS-UPDRS certified neurologists, two movement disorder nurses, two occupational therapists, one psychologist, one health-economist (Hospital Universitario of Burgos, Spain), and four computer engineers (University of Burgos, Spain).

### Study timetable

1. Pre-start procedures: January-June 2020.
2. First assessment: July 2020-December 2021.
3. Data analysis: August-September 2022.
4. Report and publications: years 2021–2022.

## Procedure

After signing the informed consent form, investigators will invite PD patients to participate in this randomized controlled trial. Patients will be randomly assigned using a block gender-randomization Excel calculator [male/female: intervention/control groups), providing an equal number of males and females with PD in the control/intervention groups (1:1). There will be no personal expenses for the participant during the study; instead, all assessments will be free, including the neurologist, nurse, psychologist, and occupational therapist evaluations. The patients will be contacted by phone to confirm they attended the treatment sessions. Likewise, reports of the assessments performed during the study will be available to improve patient adherence to the intervention protocols.

**Fig 1:**
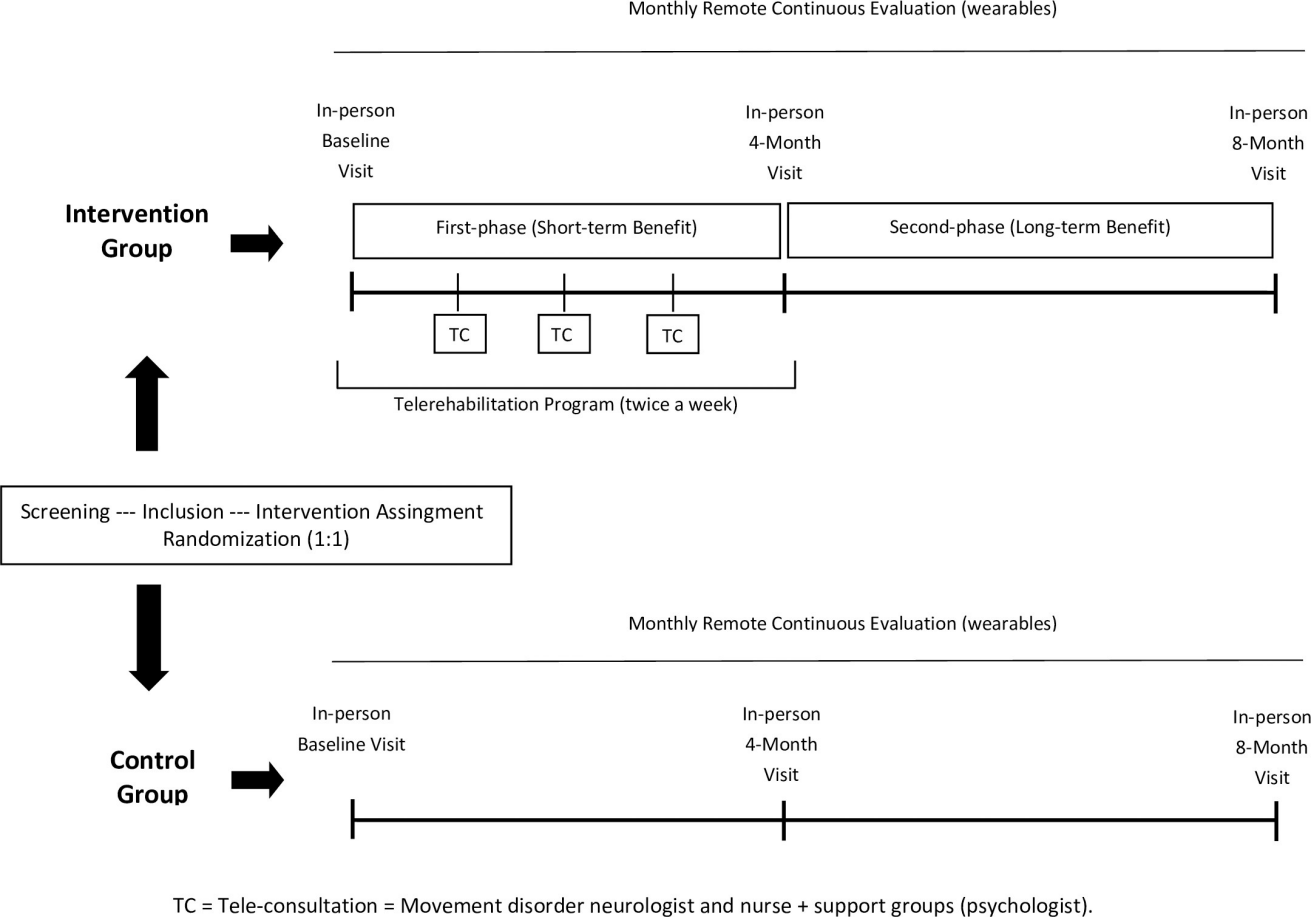
One-year, randomized, case-control, single-blinded intervention study.

Two study phases will be established (Fig 1). In the *first phase* (baseline-4^th^ month), short-term benefits will be assessed. All participants will be evaluated at baseline and four months in office. Patients allocated in the intervention group will be receiving the telemedicine fall prevention program. The occupational therapist will bring the equipment for videoconferencing to the patients’ home, set up the room for physical therapy, and evaluate the patient’s risk of performing different exercises according to the patient’s capabilities and home environment. In the *second phase* (5^th^-8^th^month), long-term benefits will be assessed. All patients allocated in the intervention group will discontinue the telemedicine program at the end of the 4^th^ month. All patients will receive standard medical care provided by the movement disorder neurologist and nurse, with a final visit at eight months from baseline.

### In-office visits (best standard medical care)

**Fig 2:**
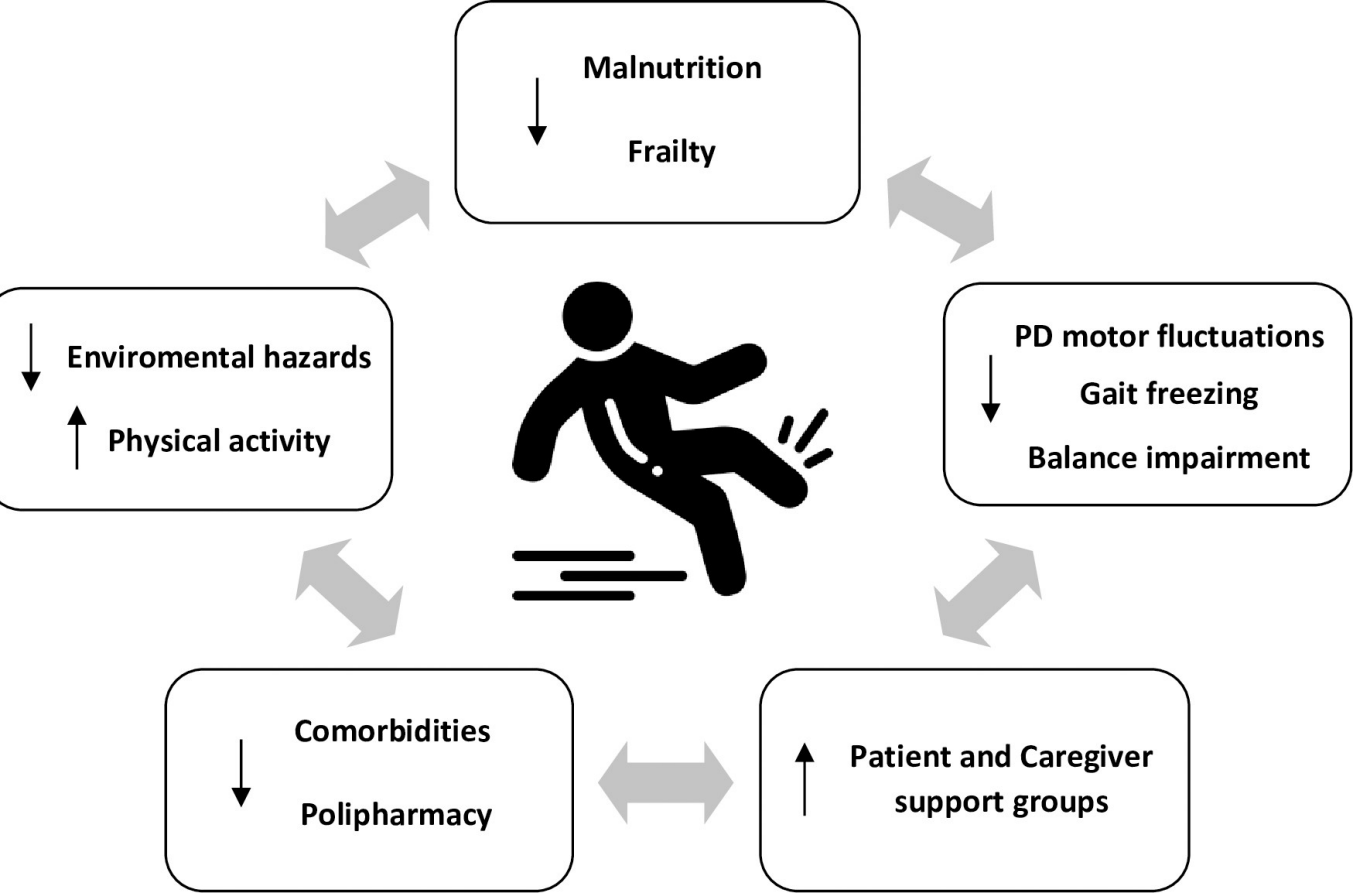
Telemedicine multidisciplinary approach for falls prevention in Parkinson´s disease.

Patients will be evaluated in-office at baseline, four months, and eight months. At baseline, we will collect information on socio-demographics (age, gender, education background, living status (urban vs. rural, alone vs. with family), and PD clinical characteristics (PD duration, drugs). In each in-office visit, we will perform a complete physical and neurological exam and will complete a standard PD follow-up visit. Consensus-based clinical practice recommendations for the examination and management of falls in patients with PD will be assessed [27]. We will evaluate vital signs (standing and lying down blood pressure), heart pulse, oximetry, falls history (number, characteristics, place), visual acuity (eye chart), nutritional and dysphagia screening, balance, and strength by dynamometry. Fall prevention will be performed by providing pharmacological and non-pharmacological therapy adjustments, education, nutritional interventions, and referrals to other physicians if needed (Fig 2).

### Virtual visits

**Fig 3:**
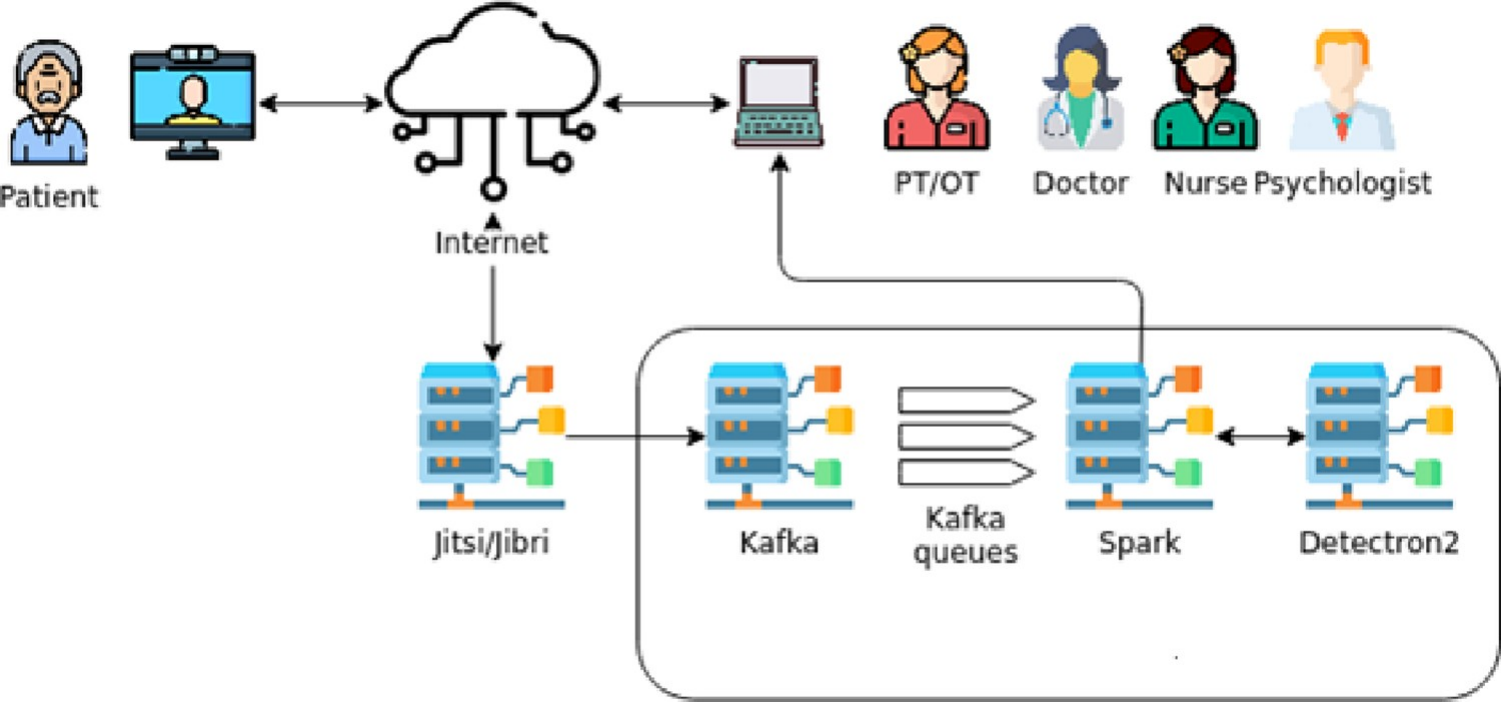
Schematic view of video data information using artificial intelligence.

Telemedicine visits in the intervention group will include telerehabilitation, neurologist and nurse telecare, and tele-support groups conducted by the psychologist (Fig 3). Tele-rehabilitation will consist of an individualized program with each of the patients, 45 to 60 minutes long. All sessions will be directed and supervised in real-time by the occupational therapist. During these telerehabilitation sessions, the occupational therapist will give the patient the necessary instructions to perform various exercises. First, a prior physical warm-up, consisting of a series of mobility exercises and stretching, followed by exercises designed to improve body posture, increase mobility, favor transfers, reduce axial stiffness, freezing, and walking disturbances, and enhancing balance and coordination, according to the cognitive, sensory-motor training therapy (Perfetti method) [28, 29]. During this cognitive sensory-motor training therapy, motor control will be improved using a specific type of repetitive sensory and motor re-learning protocol. Different exercises will be accommodated based on the patient’s capacity, including kinesthetic tasks [simple to complex parameter movement discrimination] with the corresponding cognitive activity [recognition of the direction and distance of the movement], and coordination tasks associated with executive cognitive activity [28, 29].

As a secondary objective, the occupational therapist will provide education to promote behavioral changes toward healthy lifestyles such as environment modifications, daily walks, social activities, and healthy eating behaviors. In addition, the nurse and the neurologist will conduct monthly video conferences with the patient (10–15 minutes duration each one approximately). During these visits, fall history and characteristics and medical management medications adjustments, education, nutritional interventions, and referrals if needed) will be performed. Group support interventions (fear of falling, caregiver burden) will be conducted every month by the psychologist to patients and caregivers separately.

## Study assessments

Extensive information on socio-demographic aspects, PD, comorbidity and treatment, and PD and gait dysfunction severity will be collected (Table 1). To avoid fatigue, we will recommend brief breaks to patients during assessments.

**Table 1:** Assessments.

	Screening	Baseline	4-Month Visit	8-Month Visit
Sociodemographics	✓			
Falls risk screening	✓			
Montreal Cognitive Assessment	✓			
MDS-Unified Parkinson’s Disease Rating Scale		✓	✓	✓
Hoenh & Yahr Stage		✓	✓	✓
Freezing of Gait Questionnaire		✓	✓	✓
Non-Motor Symptoms Scale		✓	✓	✓
Parkinson’s Disease Sleep Scale		✓	✓	✓
Visual Analog Scale-Pain		✓	✓	✓
Visual Analog Fatigue Scale		✓	✓	✓
Beck Depression Inventory-II		✓	✓	✓
Lille Apathy Rating Scale		✓	✓	✓
Schwab & England Activities of Daily Living Scale		✓	✓	✓
European Health Interview Survey-Quality of Life 8 item index		✓	✓	✓
Cumulative Illness Rating Scale for Geriatrics		✓	✓	✓
Cost questionnaire for patients with PD.		✓	✓	✓
Parkinson’s disease Quality of Life-39		✓	✓	✓
Malnutritional Universal Screening Tool		✓	✓	✓
Eating Assessment Tool		✓	✓	✓
European Quality of Life 5 Dimensions		✓	✓	✓
Zarit Caregiver Burden Inventory		✓	✓	✓
Mini Balance Evaluation Systems Test		✓	✓	✓
Timed Up and Go Test		✓	✓	✓
Frail Scale		✓	✓	✓
Fried Frailty Index		✓	✓	✓
Falls Efficacy Scale-International		✓	✓	✓
Telehealth Usability Scale				✓
Quebec User Evaluation of Satisfaction with Assistive Technology				✓

### In-office assessments

1) Nutritional, sarcopenia status, and frailty status: We will assess anthropometrics: body mass index (BMI) calculated by the following formula: weight (kg)/height^2^ (m^2^), screening for malnutrition with the Malnutritional Universal Screening Tool [MUST] [30], presence of dysphagia using the Eating Assessment Tool (EAT-10) [31], presence of sarcopenia based on standard bioimpedance (BC418MA, mono frequency, Tanita UK^®^, Yiewsley, UK) with the following cut-off parameters [men: severe sarcopenia with an appendicular skeletal mass < 15 kg, appendicular skeletal mass/height < 7 Kg/m^2^, women: severe sarcopenia with an appendicular skeletal mass < 15 Kg, appendicular skeletal mass/height < 5.5 Kg/m^2^] [32–34]. and handgrip strength (Jamar ^®^ Plus hydraulic hand dynamometer, cutoff for men: < 30 Kg, and for women: < 20 Kg) [35], and frailty using the Fried Frailty Index (FRIED) [36].

2) PD motor and non-motor symptoms severity will be assessed using the MDS-UPDRS [37], Non-Motor Symptoms Scale (NMSS) [38], Parkinson’s Disease Sleep Scale (PDSS) [39], Visual Analog Scale-Pain (VAS-Pain) [40], Visual Analog Fatigue Scale (VAFS) [41], Beck Depression Inventory-II (BDI-II) [42], Lille Apathy Rating Scale (LARS) [43], Schwab & England Activities of Daily Living Scale (ADLS) [44].

3) Patient’s health-related quality, caregiver burden, and comorbidities will be assessed using the Quality of life European Health Interview Survey-Quality of Life 8 item index [EUROHIS-QOL 8 item-index] [45]; the Parkinson’s disease summary index score (PDQ-39) [46], Zarit Caregiver Burden Inventory (ZCBI) [47], and the Cumulative Illness Rating Scale for Geriatrics (CIRS-G) [48].

4) Gait and postural balance impairment will be assessed using the Mini Balance Evaluation Systems Test (MINI-BEST) [49], Timed Up and Go Test (TUG and COG-TUG) [50], Frail Scale (FRAIL) [51], Falls Efficacy Scale-International (FES-I) [52], and Freezing of Gait Questionnaire (FOGQ) [53].

5) Cost of telemedicine delivery and health service use: Implementing the telemedicine program will account for the cost of equipment supplied and the average hospital wage for health personnel services. Data on health service utilization will be assessed, including direct medical costs (medical visits, hospitalization, use of other physical therapies at the end of the intervention phase, goods and services used, diagnosis, or treatment), and non-medical costs (e.g., paid caregivers, transportation, social services, paid caregivers, adaptation of accommodation, and any special equipment).

6) Satisfaction: Patient satisfaction in terms of usability of telehealth implementation and services will be measured using the telehealth usability questionnaire (TUQ) and the Quebec user evaluation of satisfaction [54, 55].

### Blinded, remote motor assessments

In all participants, we will collect waist-worn wearables data reporting On and Off states (with and without dyskinesias), gait parameters, falls and FOG using the STAT-ON™, once a month during the eight months duration of the study.

## Technology for videoconferencing and artificial intelligence

The telerehabilitation system presented in this study is composed of the server (Jitsi) through which the data are relayed, an affordable device that patients can use at home, a home television, and an additional server for applying the Artificial Intelligence (AI) techniques including Kafka, Spark, and Detectron2. All the source code of the telerehabilitation system is publicly available on Github:https://github.com/admirable-ubu/FIS-FBIS/

## Statistical analysis

### Sample size

We calculated the sample size using the Epidat^®^ 4.1 statistical software. Considering that a 50% fall reduction in the number of falls compared to baseline will be obtained in 24% of the participants in the intervention group and 2% in the control group, we estimated that 70 patients (35 per group) would provide 80% power (5% probability of type 1 error) with a confidence level of 95%. By assuming a 9% dropout rate, a total sample of 76 patients will be then included in this study.

### Outcome measures

The study’s primary outcome measure will be the total number of falls in the 4-month (short-term benefit) and 8-month (long-term benefit) after randomization. A fall will be defined as unintentionally coming to rest on the ground or other lower surface without overwhelming external force or a major internal event [56].

Other secondary outcome measures will include: 1) Cost-effectiveness, which will be estimated using natural units of health outcomes, including the incremental cost-effectiveness ratio per fall prevented and cost per extra person avoiding mobility deterioration (identified as an average number of daily steps assessed by wearable sensors); 2) A cost-utility analysis using the EuroQoL will estimate the incremental cost per quality-adjusted life-years (QALY) gain; 3) Short and long-term rate of change from baseline to four and eight months, respectively of motor, NMS, gait, balance, HR-QoL, caregiver burden, and comorbidities severity; 4) Feasibility (completed telemedicine visits) and adherence (dropout rate of telemedicine visits).

### Statistical analysis

The study includes several necessary methodological resources that will minimize the risk of bias, such as the randomization process, allocation concealment, blinded assessment of the results, and intention-to-treat analysis. A statistician blinded to group allocation will supervise the data entry and analysis. The normality of the variables will be evaluated using the Kolmogorov-Smirnov test and graphs, including histograms and normal distribution plots. For the description of the quantitative variables, the mean and the standard deviation will be used, and for categorical variables, the frequency distribution and percentages. For non-normally-distributed variables, we will use the median, the interquartile range, and the corresponding non-parametric tests. The association between independent categorical variables will be analyzed using the χ2 test or Fisher’s exact test. The means between the two groups will be compared using the one-way analysis of variance (ANOVA). We will analyze the relationship between quantitative variables using correlation coefficients.

We will use logistic regression models to analyze the relationship between fall strategy prevention of 50% reduction in the incidence of falls (yes vs. no) as the dependent variable with the intervention vs. the control group, as the independent variable adjusted for age, gender, total motor MDS-UPDRS, comorbidity severity, and total levodopa equivalent dose. Other pertinent univariate and multivariable analyses will be performed based on the objective type. Also, given the complexity of potential analysis, including a diversity of variables from different origin and measurement properties, advanced statistical methodology (data mining, AI techniques) will be applied as needed. The level of significance will be set at p < 0.05. Statistical analysis will be performed with SPSS V.25.0. (IBM SPSS Statistics for Windows, Armonk, NY, USA).

## Future technological possibilities

The big data architecture used in this paper will ensure maximum data transference rates for immediate communication and ease of use. The design of the big data architecture will be based on the Extract, Transform, and Load (ETL) process [52]. Videos will be obtained during the video conference connection with the occupational therapist, anonymized using a 3×3 Gaussian filter (to avoid recognizing the patient). Body skeleton models will be created using deep neural networks and will be trained with the Common Objects in Context (COCO) image dataset [56]. The occupational therapist will use the patients’ skeleton information to evaluate whether the exercises are properly performed. Moreover, with this information, the implementation of AI could detect the patients’ improvements over time.

## Discussion

The purpose of this study will be to analyze the additional benefit of establishing a multidisciplinary telemedicine fall prevention program for patients with PD. Despite evidence of the effectiveness of a multidisciplinary approach for PD falls reduction, there is a need to integrate what is known in theoretical models and what still needs to be done for health behavior changes. We will be providing a multidisciplinary telemedicine intervention in the individual’s environment, where the falls usually happen, adding the possibility of tailoring the treatment to their environment. It is well known that rehabilitation is cost-effective compared with usual care [57], and in this regard, telerehabilitation has likewise emerged as a good opportunity, reducing postural instability [58] with a low rate of dropouts [59]. However, in terms of fall reduction, the largest randomized controlled trial conducted in 474 patients with PD demonstrated that rehabilitation was not effective in reducing repeat falling in the trials, in contrast to other functional tasks and self-efficacy, suggesting differential patterns of intervention [60]. To our knowledge, there is no published data on the cost-effectiveness of a multidisciplinary telemedicine team approach for fall prevention in PD and the duration of such benefit (short-term vs. long-term benefit).

Currently, several applications of telemedicine for PD have been adopted. Many studies have applied synchronous and remote follow-up visits to enable face-to-face virtual interaction and increase patients’ access to specialized health care [61]. For patients with PD, those who see a movement disorder specialist live longer, receive higher-quality care, and maintain a better standard of living, which shows those who merely see a primary care physician or a general neurologist [62, 63]. On the other hand, multidisciplinary care is extremely important for treating people living with PD. With our increasing knowledge on the complexity and heterogeneity of symptoms that people with PD can experience, the need for an integrated, patient-centered multidisciplinary approach is gaining importance. Specific healthcare disciplines, such as the movement disorder neurologist, nurse specialist, and physiotherapist, and occupational therapists, are already considered essential multidisciplinary team members [8]. However, a multidisciplinary approach is usually limited due to extraordinary circumstances such as the Covid-19 pandemic, limited availability of healthcare provider personnel (waiting list, underserved areas), and patient’s limited access due to costs, mobility problems, long-distance traveling, and lack of available caregivers. In this regard, there is no doubt that the results of this study could be important for health policymakers to reorganize licensed health personnel resources to create telemedicine multidisciplinary PD care teams without geographical limitations, allowing equity in the distribution and access to specialized health care.

In addition to rehabilitation and the optimal pharmacological management in PD, there is a growing interest in the impact of nutrition in reducing the risk for frailty and secondary falls. The combination of nutrition, sarcopenia, frailty, and rehabilitation data could be used to provide patient-centered motivational, educational strategies [64] to reduce sedentary lifestyles and to delay mobility deterioration, which represents further originality.

Additional features of this novel program will include the use of wearable sensors and AI. Using these new technologies, we will assess the video-based physical exercise performance to assist the physical therapist, and we will obtain ecologically, rater-independent outcomes of motor symptoms and physical activity using wearables validated algorithms for PD during the intervention and follow-up study phases [65].

The potential critical aspect of our study is the exclusion of PD patients with moderate-severe cognitive impairment. Whereas adults with cognitive problems are one of our society’s most vulnerable sectors and more likely to experience falls, preserved cognitive skills are considered critical to delivering telemedicine care and rehabilitation. Given the characteristics of the PD population, other critical challenges include feasibility aspects in patient retention and adherence to this study.

In conclusion, this study is a challenging and original initiative. We hope this project will provide important information regarding the cost-effectiveness of a multidisciplinary telemedicine fall prevention program for PD patients who are not currently benefiting from these strategies. All results from the study will be communicated by publication without any restriction.

## Supporting information

S1 Checklist CONSORT 2010. Description: checklist of information in this randomized trial.
(DOCX)

S2 Checklist Spirit. (DOC)

S1 File Original research protocol. (DOCX)
