# Supplementary material for: Adopting a multidisciplinary telemedicine intervention for fall prevention in Parkinson’s disease. Protocol for a longitudinal, randomized clinical trial
Source: PLoS One. 2021 Dec 21;16(12):e0260889. doi: 10.1371/journal.pone.0260889 (PMC8691608; doi:10.1371/journal.pone.0260889)
Supplement: S1 File — (DOCX) [file pone.0260889.s003.docx]

**TITLE:** Feasibility and Cost-Effectiveness Study of Telemedicine Use with a Multidisciplinary Team for the Prevention of Fallout in Parkinson's Disease

**MAIN RESEARCH:** Esther Cubo

**MAIN CO-RESEARCHER/A:**


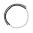

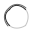

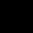

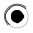


| **PROJECT TYPE:** | **INDIVIDUAL** | **COORDINATED** | **MULTICETRIC** |
| --- | --- | --- | --- |
| **IP COORDINATOR NAME:** | |  |  |
| **(Complete only for coordinated projects)** | | |  |
| **DURATION:** | **3 YEARS** |  |  |
|  | | |  |
| **SUMMARY (Project Objectives and Methodology)** | | | **(Fit to available space)** |


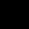

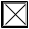


Falls in Parkinson’ s disease (PD) are very common with devastating consequences, increasing comorbidity, mortality, and social health cost. Since patients with PD have limited access to multidisciplinary intervention, this study wants to evaluate from a cost-effective point of view, new forms of healthcare using telemedicine, and use of fall sensors in order to reduce the incidence of falls in PD. This study will be multicenter, longitudinal, randomized, including 76 patients with idiopathic EP, without cognitive impairment and at high risk of fall: 38 (PD controls) using the best usual clinical management in the consultation, 38 (intervention group) using tele-rehabilitation and teleconsulta with the neurology service), for 9 months.

The main efficacy variables will be the comparison of the incidence of falls from baseline and the cost-effectiveness (Incremental cost-effectiveness ratio by reduction of falls and QALYs). The results of this study will allow to study the feasibility of remote healthcare to prevent falls in patients with PD, allowing equity and access to the best care in a specialized unit.

**TITLE:** Feasibility and cost-effectiveness of a multidisciplinary home-telehealth management program to reduce falls in Parkinson's disease.

**ABSTRACT (Objectives and Methodology of the Project) (Please only use the space provided below)**

Falls in Parkinson´s Disease (PD) are very much suffering from devastating consequences, mortality, quality of life and increased socio-health costs. However, despite being an area of priority in the therapeutic management of these patients, there are no guidelines for programs to prevent in PD. Given that socio-sanitary object is limited, and patients with PD have limited access to multidisciplinary, this multicenter, multidisciplinary study aims to evaluate, from a cost-effective point of view, new forms of healthcare using telemedicine, and the use of sensors in order to prevent the occurrence of falls in PD. This study will be a multicenter, longitudinal, randomized study where patients with idiopathic PD will be included, without cognitive improvement and with high risk of falling. Two study groups will be followed during 9 months at the Movement Disorders Unit (Hosp. Univers. Burgos): 38 patients with PD (controls) office-based care with standard clinical management, and 38 patients (intervention group) using tele-and teleconsultation.

This study will analyze as main outcome variables: the comparison in the incidence of falls with respect to the baseline situation and the cost-effectiveness between the two groups of participants, including the Incremental cost effectiveness ratio for falls and QALYs during the study. The results of this study will allow us to study the feasibility of remote health care to prevent patients in patients with PD, allowing equity in the distribution and access to specialized health care.

11, 12

**File No.**

**MAIN RESEARCH: Esther Cubo**

**REQUEST MEMORY HEALTH RESEARCH PROJECT**

**BACKGROUND AND CURRENT STATUS SECTION OF THE TOPIC**

**Purpose of the project, background and current state of scientific-technical knowledge, national or international groups working in the specific line of the project or in related lines.**

**Mention references in the following section: Most relevant literature. Maximum 3 pages (15,700 characters)**

**Falls** in Parkinson's disease (PD) are more frequent compared to healthy controls from the community,^1 with^ often devastating consequences. Historically, it was believed that falls represented a late manifestation of PD due to the progression of axial symptoms combined with aging.^2^ However, although true, falls are not exclusive in elderly or elderly people with advanced PD. In a recent study, one or more recent falls were observed in patients with PD below 50 years of age,^3^ and even the risk of hip fracture increased even 15 years before the development of EP.^4^ Fall in PD is then associated with a worse prognosis, comorbidity, and mortality.^55^ In terms of progression of EP, existence Falls, worsens the clinical management of these patients, increasing institutionalization, and ultimately reduces survival in EP.6. Falls also produce a significant psychological impact, developing up to 40% of people who fall or are at risk of falling into a syndrome of fear of falls, which increases the confinement of these people in the home and in the physical activities.com7 Finally, the reduction of physical activity in people with frequent falls, increases sedentarism, with a reduction of muscle mass and cardiovascular risk, the occurrence of peripheral vascular complications with the appearance of ulcers, and osteoporosis (which in itself increases the risk of fractures).8 All these consequences lead these people to a situation of greater dependency, isolation, institutionalization, decrease of quality of life, socio-health cost increase^.9, 10^

**Fall prevention:** The comorbidities and functional impact of falls make the prevention of falls in PD a top priority area in the therapeutic management of these patients. However, there are no guidelines or programs in place to prevent falls in EP. In general, prevention programs should take into account the peculiarities of PD such as unpredictable pharmacological responses, gait blockages, associated cognitive impairment, but also the potential factors associated with falls that we find in the general population, such as polypharmacy, and the existence of osteoporosis. Different studies have observed that there are at least 30 risk factors associated with a higher risk of falls in PD, and although not all of them are modifiable, only some of them have been included in the therapeutic guidelines for the prevention of falls.^13^ Below we describe the published interventions for the prevention of falls: (1) Education: Different protocols have proposed various strategies such as reducing risk factors in the home (modification of furniture in the bathroom, etc.), restricting alcohol consumption, avoiding the use of polypharmacy, hydration for diminishing orthostatic hypotension, modification of lifestyles promoting physical exercise, etc. 2) Exercise-based interventions: Although there are contradictory data on the effectiveness of the exercise in the prevention of falls, in the most recently published meta-analysis, 5 short-term studies (620 participants) and 6 long-term studies (590 participants) have been included, demonstrating efficacy immediately and during follow-up.^14^ One of the most important factors to consider in the prevention of falls is the interaction between the severity of EP and efficacy of physical therapy. It has been observed that patients with less severity of motor symptoms (UPDRS motor score <26) had a reduction in the incidence of falls of 69% (incidence rate ratio 0.31, 95% CI 0.15-0.62, P<.001), while those with a motor UPDRS score > 27, they showed an increase of 61% of falls (incidence rate ratio 1.61, 95% CI 0.86-3.03, p=0.13).^15^ Non-pharmacological interventions in PD have also been shown to be effective in increasing the quality of life of patients with PD. A recent meta-analysis reviewing 26 studies on the efficacy of non-pharmacological interventions to improve the quality of life of patients with PD, objective evidence of class I that intervention through physical exercise improved the quality of life of patients with PD.^16^ Also, there is preliminary evidence that interventions based on minimally supervised physical rehabilitation programs designed to reduce risk factors (decrease of balance, loss of strength In those patients with less severity of PD, it is cost-effective.^15, 17^ In a randomized international study of patients with PD (cognitive intervention through virtual reality plus the use of physiotherapy + physiotherapy) vs. the use of physiotherapy alone,^18^ with a similar baseline incidence of falls in both. groups (11.9・+39.5 falls per 6 months), only those patients who had combined cognitive intervention and physical physiotherapy had a significant reduction in the incidence of falls after 6 months

compared to baseline [6.0 drops (95% CI 4.3-8.2), p<0・0001], and this improvement remained 6 months after the end of the intervention [ 0.5 drops, 95% CI 0.3-0.9; p=0.03).^18^

1. Pharmacological Interventions: The usual clinical practice includes 1) the management of general risk factors for falls such as osteoporosis treatment, orthostatic hypotension, and 2) strategies to decrease specific risk factors in EP such as Off periods (without benefit of dopaminergic medication) and gait blockages (usually more frequent during Off periods than on (under the benefit of anti-Parkinsonian medication)], or peak dose dyskinesias due to motor complications, following guidelines established in the pharmacological/surgical treatment for PD (See protocol in supplementary material). To date, few pharmacological interventions have been published exclusively designed to improve the gait and risk of fall in the EP. In a randomized double-blind control study, the efficacy of an acetylcholinesterase inhibitor, rivastigmine (3-12 mg) was studied in 355 patients with PD. An improvement of 45% was observed after 32 weeks, due to an improvement in the reduction of falls and speed of gait, in single gait and in dual-action gait.^19^ This benefit on the fly could therefore be attributed to an improvement in cognitive (executive) function. Another study analyzing patients with PD and symptoms of neurogenic orthostatic hypotension, a goal that droxidopa 300-1800 mg/day, for 10 weeks, improved by 0.4 falls/patient/week compared to 1.05 falls/patient/week in the placebo group.^20^ This cost-effective improvement was achieved, attributed to the decrease in orthostatic hypotension, without impact on motor signs of PD, clinically measured by UPDRS.^21^
2. Access of patients to non-pharmacological health interventions: The clinical practice and usual management of patients with PD consists of education, and pharmacological interventions provided in Neurology consultations (nursing and neurologist). Non-pharmacological interventions such as speech therapy, occupational therapy, physiotherapy, and cognitive are generally provided in patient associations, private centers, geriatric homes, or minimal hospital care centers. **It is estimated that only 20% of patients with PD benefit from multidisciplinary therapeutic interventions in Spain (source: Spanish Parkinson Federation)**. **Therefore, the access of chronic patients, in this case the EP to units of motion disorder, and multidisciplinary equipment, is not equitable for all patients**. Geographical variability (rural, urban), university hospital centers vs. comarcal hospitals, etc., and transport problems (need of a family member), ambulances, make there a situation of inequality in healthcare for this population. Numerous studies have been published showing that telemedicine-provided health care (synchronous=videoconferencing, deferred =asynchronous), is similar to the standard care provided in the consultation, allows for significant savings in time and economic cost from transporting patients (usually in charge of patients), facilitating access to specialized consultations with neurologists, and other health professionals.^22^

**Fall assessment**. The simplest method to detect falls is to ask the patient if they have fallen during clinical consultation. However, forgetfulness may exist, especially in patients with associated cognitive impairment who have unseen falls. Therefore, the great challenge is to be able to find measures that can predict and evaluate the risk of falls in patients with PD. In order to predict the risk of falls, different scales have traditionally been used: equilibrium, gait, and functional mobility maneuvers (Berg balance scale, Dynamic Gait index, Timed Up and Go), but the big disadvantage is that most have used cross-cutting designs that differentiate us only from those that don't fall, without evaluating risk prospectively. Recent studies have published: 1) the equilibrium assessment test (Best, and mini-Best) that have incorporated the different previous scales into a battery, establishing a cut-off point of 20/32 points that predicts the risk of fall in the next 6 months with an area under the curve of 0.87, sensitivity of 0.86, and specificity of 0.87.^23^ There is also a specific questionnaire for EP, which with 3 items, establishes a risk of falls in the next 6 months in low, moderate, high predictive validity of 83%, sensitivity of 91%, but with a specificity of 66% in patients with PD and cognitive impairment, and is then recommended to evaluate the risk of falls in PD (supplementary material).^13^ Recently the devices called sensors that the patient, based on inertial systems (accelerometers, gyroscopes), can evaluate different gait patterns, On vs. Off states, and even falls. The great advantage of these devices is the ability to measure objective gait data in real environments (outside the hospital), and potentially identify those patterns (algorithms) that can predict the risk of falls. A pilot study showed that objective gait pattern data (cadence, length of step, blockade) can better predict the risk of fall than the assessment maneuvers performed in gait laboratories, even among patients who had not previously fallen.A Spanish team led by Joan Cabestany have developed a sensor (STAT-ON) that can discriminate motor states On vs. Off , i.e. motor fluctuations in EP; and gait disorders with a sensitivity of 98% and a specificity of 88% (see references in supplementary material)

In summary, although the prevention of falls is a social and health priority, the cost-effective strategies in the EP that allow detecting the risk of falls and preventing them have not been thoroughly studied. Given that the social and health resources are limited, this multi-center, multidisciplinary study wants to evaluate the cost-effectiveness,

new forms of healthcare using telemedicine, and use of fall sensors in order to reduce the incidence of falls in EP.

**File No.**

**MAIN RESEARCH: Esther Cubo**

**REQUEST MEMORY HEALTH RESEARCH PROJECT**

**BACKGROUND AND CURRENT STATUS SECTION OF THE TOPIC**

**Refer to references in the preceding paragraph: Background and Current Status. (Maximum 1 page)**

1.Pickering RM, et al. A meta-analysis of six prospective studies of falling in Parkinson's disease. Mov Disord 2007;22:1892-1900.

2.Kempster PA, et al. Patterns of levodopa response in Parkinson's disease: clinical-pathological study. Brain 2007;130:2123-2128.

3.Voss TS, et al. Fall frequency and risk assessment in early Parkinson's disease. Parkinsonism Relat Disord 2012;18:837-841.

4.Nystrom H, et al. Risk of Injurious Fall and Hip Fracture up to 26 and before the Diagnosis of Parkinson Disease: Nested Case-Control Studies in a Nationwide Cohort. PLoS Med 2016;13:e1001954.

5.Coughlin L, et al.Fractures in patients with Parkinson's disease. Clin Orthop Relat Res 1980:192-195.

6.Wenning GK, et al. Progression of falls in postmortem-confirmed parkinsonian bacteria. Mov Disord 1999;14:947-950.

7. Grimbergen YA, et al. Impact of falls and fear of falling on health-related quality of life in patients with Parkinson's disease. J Parkinsons Dis 2013;3:409-413.

8.Speelman AD, , et al. How might physical activity of patients with Parkinson disease? Nat Rev Neurol 2011;7:528-534.

9. Muslimovic D, et al. Determinants of disability and quality of life in disease to Parkinson disease. Neurology 2008;70:2241-2247.

10.Grimbergen, et al. Gait, postural instability and seizure. In: Olanow CW, editor. The non-motor and non-dopaminergic features of Parkinson's disease. John Wiley and Sons ltd; 2011.

11. Gillespie LD, et al. Interventions for preventing falls in older people living in the community. Cochrane Database Syst Rev 2012:CD007146.

12.Kerr GK et al. Predictors of future falls in Parkinson disease. Neurology 2010;75:116-124.

13.Fasano A, et al. Falls in Parkinson's disease: A complex and changing picture. Mov Disord 2017;32:1524-1536.

14.Shen X, et al. Effects of Exercise on Falls, Balance Sheet, and Gait Ability in Parkinson's Disease: Meta-analysis. Neurorehabil Neural Repair 2016;30:512-527.

15. Canning CG, et al. Exercise for falls prevention in Parkinson disease: to randomized controlled trial. Neurology 2015;84:304-312.

16.Ahn S, et al. Effects of Non-Pharmacological Treatments on Quality of Life in Parkinson's Disease: A Review. J Parkinsons Dis Alzheimers Dis 2017;4.

17. Farag I, et al. Economic evaluation of a falls prevention program among people With Parkinson's disease. Mov Disord 2016;31:53-61.

18.Mirelman A, et al. Addition of a non-immersive virtual reality component to treadmill training to reduce fall risk in older adults (V-TIME): to randomized controlled trial. Lancet 2016;388:1170-1182.

19.Henderson EJ, et al. Rivastigmine for gait stability in patients with Parkinson's disease (ReSPonD): a randomized, double-blind, placebo-controlled, phase 2 trial.

Lancet Neurol 2016;15:249-258.

20.Hauser RA, et al. Droxidopa and Reduced Falls in a Trial of Parkinson Disease Patients With Neurogenic Orthostatic Hypotension. Clin Neuropharmacol 2016;39:220-226.

21.Francois C, et al. Cost-effectiveness of droxidopa in patients with neurogenic orthostatic hypotension: post-hoc economic analysis of Phase 3 clinical trial data. J Med Econ 2016;19:515-525.

22. Beck CA, et al. National randomized controlled trial of virtual house calls for Parkinson disease. Neurology 2017;89:1152-1161.

23.Mak MK, Auyeung MM The mini-BESTest can predict parkinsonian recurrent fallers: 6-month prospective study. J Rehabil Med 2013;45:565-571.

24. Postuma RB, et al. MDS clinical diagnostic criteria for Parkinson's disease. Mov Disord 2015;30:1591-1601.

**File No.**

**MAIN RESEARCH: Esther Cubo**

**REQUEST MEMORY HEALTH RESEARCH PROJECT**

**HYPOTHESIS AND OBJECTIVES SECTION**

**(Fit to available space)**

**HYPOTHESIS**

1. Falls in EP have a multifactorial etiology secondary to: a) factors specific to PD (infraction of motor symptoms, gait blockages, treatment complications, dyskinesias, motor and non-motor fluctuations), b) generic such as aging itself (osteoporosis, cognitive impairment), polypharmacy, comorbidities: neuropsychiatric disorders (anxiety, depression), c) environmental factors (risks in the home, pavements, etc.)
2. The risk of falls and secondary comorbidities in patients with PD can be prevented through improved fall detection systems (sensors) and through multidisciplinary intervention that modifies potentially treatable risk factors
3. Telemedicine is a cost-effective assistance tool for the prevention of falls, especially in patients with limited access to non-pharmacological interventions and specialized units of motion disorder.
4. Multidisciplinary intervention in patients with PD and risk of fall can produce additional benefits, with an impact on the evolution of the disease (motor and non-motor symptoms), and quality of life of the patient and caregiver load.
5. Multidisciplinary intervention in PD can produce long-term effects on the risk of falls by changing lifestyles in the patient, health status and environmental risks.

**OBJECTIVES**

1. General:
   1. To analyze the cost-effectiveness of a multidisciplinary intervention composed of neurologists, nursing, rehabilitation (physiotherapists, occupational therapists, psychologist), through telemedicine, to reduce the risk of falls and secondary comorbidity compared to the usual clinical practice (ambulatory consultation in a Unit of Movement Disorder).
2. Specific
   1. To study the feasibility, difficulty, satisfaction, adherence to a health intervention through telemedicine (videoconferencing) and use of new technologies (motion sensors).
   2. To study the best method that allows us to detect and predict falls in patients with PD (screening questionnaires, vs. MDS-UPDRS clinical scales, vs. daily falls of patients, vs. falls sensors and gait disorders).
   3. To design an ecological intervention protocol for the prevention of falls, valid for outpatients with PD and limited access to non-pharmacological therapies.
   4. To compare the burden of caregiver and progression of motor, non-motor symptoms, quality of life related to the health of patients with PD, who receive a multidisciplinary intervention and residual benefit after the end of the intervention, vs. the usual clinical practice at the level of the neurology consultation (Unit of Disorders of the Movement).

**File No.**

**MAIN RESEARCH: Esther Cubo**

**REQUEST MEMORY HEALTH RESEARCH PROJECT**

**METHODOLOGY SECTION**

**Design, study subjects, variables, data collection and analysis and limitations of the study.**

**Maximum 3 pages (15,700 characters)**

Design: pilot, longitudinal, case-control, randomized, unilateral blind study.

Sample of patients: consecutive outpatient patients diagnosed with idiopathic EP (MDS criteria for EP),^24^ older than 18 years of age, with Hoehn Yahr stadiums <3, with a motor score at UPDRS motor scale < 27,15 with an MSE>MOCA 151. 8, and a score > 8 in the fall screening questionnaire (CribC). Patients who are unable to walk independently for at least 5 minutes, with severe psychiatric pathology, cerebrovascular disease, severe cranial trauma, severe orthopedic problem in lower limbs or spine, peripheral neuropathy, rheumatological or other serious systemic diseases (cancer, etc.), sensory deficits which the investigator believes may affect the development of the research, and who are currently performing therapies, shall be excluded non-pharmacological through patient associations and other centers.

Ethics: All patients and caregivers sign informed consent prior to the patient's inclusion in the study. This study will be approved by the Ethics Committee of the participating centers. All clinical information will be included in protected databases in accordance with the current data protection law.

Randomization: A computerized randomization will be performed. Two study groups will be established. 1) Group A

(intervention): Patients with PD + multidisciplinary intervention through telemedicine; Group B (Control):

Patients with PD + follow-up following the usual clinical practice in Neurology consultation.

Blind assessment:Blind, objective evaluation of the severity of the motor symptoms, motive states ON (response to dopaminergic medication), OFF (non-response to dopaminergic medication), gait blockages, falls, shall be done through the motion sensor placed in the waist. The sensor, commercially called STAT-ON, is a portable medical device inserted into a specific belt. The system is integrated with a set of algorithms to evaluate the walking parameters, such as walking fluidity, number, length and speed of steps. The amount of movement, minutes walking and the number of postural transitions can also be monitored. All algorithms have been validated with scientific studies (references in the supplementary material). The sensor is flexible and adaptable to any platform and can be easily read with a mobile app, which generates a report with the information obtained in the study days at the disposal of the neurologist. These sensors will be carried by all the participants in the study. In the intervention group (A): will be used for recording falls, motor status, gait disorder. This information will feed-back to the neurologist in order to use therapeutic strategies. In Group B, the clinical information will be collected at the end of the study and will serve only as an objective record of the above parameters.

Video conferencing: this synchronous connection will be developed by the University of Burgos (confidential, secure and in accordance with the current Organic Law on Data Protection). This development will make it possible to convert the patient's television into a SmartTV using a low-cost device, quantitatively capture the patient's movements (consoles), and posture estimation by image analysis.

**Procedure:**

This project will involve three centers, the University Hospital of Burgos (responsible for pharmacological intervention), the Faculty of Health Sciences and the Computer Engineering School of the University of Burgos (responsible for non-pharmacological intervention, and teleconferences, respectively).

Evaluators & Pilotage & Training: A pilot will be conducted prior to the start of the project on the start-up of Smart TV and the establishment of videoconferencing and the launch of the tele-rehabilitation package; and training for the positioning of the gait sensor, and clinical interpretation of the reports obtained through the sensors. Evaluations with validated clinical scales and certified training will be performed for those that are needed. All patients included in the study will receive training on how to complete the weekly drop-off journal during their participation in the study (as per digital daily settings, paper journal, Smartphone app).

Evaluations: The following evaluations will be performed in all patients included in the study. There will be 4 visits in the hospital center: at baseline, 3 months, 6 months and 9 months, and in group A (intervention) there will be additional weekly (tele-rehabilitation) and monthly (nursing and neurology) remote visits. During the 9 months of participation in the study, participants will carry the gait/fall disorder sensor, and complete weekly drop-down diaries.

1) Baseline visit. At the hospital consultation. *Group A (intervention) and B (control).* Collection of demographic data, years of education and previous knowledge on technology, clinical data (PD), pharmacological treatments (equivalent dose of levodopa, other drugs), retrospective information on the number of falls in the last 6 months, Body Mass Index (BMI), blood pressure (evaluation protocol for determination of neurogenic orthostatic hypotension), presence of dysphagia (volume-viscoviscosis test) density) and malnutrition screening test (MUST); severity of motor symptoms: MDS-UPDRS, Hoehn Yhar Stadium, ; March freezing scale (FOG), CribC questionnaire; Quality of life: PDQ-39, EuroQo-5Q, Non-Engine Symptoms. NMS questionnaire, Apathy Evaluation scale (AES), cognitive function (PD-CRS), Anxiety and Depression (HADS scale), Impulse control disorders (shortened Quip scale), comorbidity (CIRS-G), caregiver burden (Zarit caregiver burden), structured questionnaire of social health resources over the last 6 months [direct medical costs (drugs, consultations, income. ...), non-direct (transport, caregiver, etc). In addition, the occupational physiotherapy and therapy group will carry out the following evaluations (additional text material).

Subsequently, pharmacological changes and appropriate educational measures will be established in all patients following evaluation by the neurologist and nurse of the consultation: nutritional, dysphagia prevention, orthostatic hypotension control etc (Falls Task Force protocol, supplementary material). All patients will be provided with a brochure about falls prevention, home safety measures and home exercises. All patients included will carry the sensor, to be placed every day, the maximum number of hours while awake and will be instructed to perform a weekly diary.

*Group A (intervention)*: Months 0-3: During the basal visit, they will be trained on how to conduct videoconferences, and a technician will be in charge of establishing the necessary connections on TV, cameras, in the home of these patients. On a weekly basis, the group of tele-rehabilitation will carry out interventions during the ON state of the patient (additional text material). Once a month, the nurse at the clinic will conduct a videoconference with the patient and caregiver, with hesitation resolution, education, general health measures, and the administration of the falls questionnaire. Every month, neurologists will receive a monthly report provided by the sensor, which summarizes the number of falls, locks/gear quality and on-off motor states. According to the clinical information provided by the sensors and the information provided by the nurse, the neurologist will have the option of performing a videoconference and performing the necessary therapeutic interventions according to the usual clinical practice in the EP. All videoconferences will be held on a regular day from Monday to Friday, with the University of Burgos providing technological support for their operation.

Visit 3 months: At the hospital consultation. Groups A (intervention), B (control), data of diaries of falls, BMI, blood pressure measurement (orthostatic hypotension protocol), and administration of MDS-UPDRS, and FOG will be collected. Based on the information obtained by clinical examination obtained in consultation only (Group B), and clinical information in consultation + monthly information obtained by sensors (Group A), the necessary therapeutic adjustments will be made based on standard clinical practice.

Group A (intervention): Months 3-6: A protocol similar to the previous one (0-3 months) will be established.

Visit 6 months: At the hospital consultation. Groups A (intervention) and B (control). The same clinical information and questionnaires collected during the basal visit will be collected. Appropriate therapeutic adjustments should be made. In Group A (intervention), information will be collected through semi-structured and analog questionnaires of satisfaction, technological difficulties on the part of patients and health professionals, and incidences (technological, medical). In Group B (control) satisfaction questionnaires.

Visit 9 months. Final study. At the hospital consultation. The protocol for the clinical information collection visit will be equal to the baseline visit and 6 months. All sensors will be collected.

**Analysis**

Sample size: For the calculation of the sample size we have based on the publication where the “minimum clinically important difference" for an intervention to prevent falls in EP with a 50% reduction in the incidence of falls. With a confidence level of 95%, a statistical power of 80%, an estimated loss percentage of 10% and considering that the reduction of falls will be effective between 60%-25% of patients in group A (intervention), and 2% for controls, we would need between 11-38 participants in each group, opting for the most conservative size, 38 participants by group.

**Measures**

Main measure of effectiveness: 1) *Difference in incidence of falls* during 6 months of study between groups A and B. Incidence = number of falls from baseline to 6 month visit, provided by patients’ diaries, and fall sensor; 2) *Cost effectiveness*. International measures will be used: (a) *QALYs*: years adjusted for quality of life, value of 0 (death)-1 (perfect health): calculated with the transformation of EuroQoL EQ-5D values into previously validated utility values in the Spanish population. (B) *Total costs* (medical direct + non-medical) in euro, during the study period. (C) Incremental cost effectiveness ratio (*ICER*): calculated as c.1: Total costs (intervention group)-Total costs (control group) / total number of falls (intervention group)-total number dropped (control group). This measure will provide us with the necessary expenditure to reduce a drop in the intervention group; c.2: Total costs (intervention group)-Total costs (control group) / QALYs (intervention group)-QALYs (control group). This measure will provide us with the necessary expenditure to increase 1 QALY.

Secondary measures of effectiveness:

1. *Severity of EP and progression*: Comparison of clinical score differences between final visit 6 months less baseline visit will be calculated for all clinical scales for motor symptom severity (MDS-UPDRS, FOG, CribC), non-motor symptoms (SNM, AES, Quip, PD-CRS, HADS), quality of life (EuroQoL, PDQ-39), balance (Mini-Battery Mini-Battery and BMI, between groups A (intervention) and B (control).
2. *Fall Detection*: Comparison of the incidence of falls provided by diaries of falls (nurses, patients) vs. information provided by sensors in the total group of participants in the study.
3. *Feasibility in the use of telemedicine*: Number (%) completing the study, adherence to tele-rehabilitation

(%) (number of sessions programmed/completed), adherence to sensors (%) (number of days patients use sensors/number of days the patient participates in the study), satisfaction and patient/professional difficulty (analog scales 0 to 10=maximum satisfaction/maximum difficulty), technological incidents (description of technological failures), safety (medical complications occurring during tele-rehabilitation).

1. *Residual benefit on the incidence of falls (lifestyle modification):* We will compare the incidence of falls between visits 6 and 9 months, and the differences in the severity of EP measured by the different clinical scales between baseline visits, 6 months and 9 months between groups A (intervention) and B (control).

Statistical analysis: The following statistical software will be used: SPSS-IBM version 21, Epidat version 3.1 software (Galician Health Service), SAS (free statistical software). A pre-project statistical analysis plan will be established with the intention of addressing. Cases, scores of lost clinics will not be imputed. The normal distribution of interest variables (Kolmogorov-Smirnov test) will be analyzed. Descriptive analyzes (mean +standard deviations, medium (interquartile ranges), frequencies (percentages), with confidence intervals of 95% with the socio-demographic and clinical variables of efficacy will be established. In order to analyze the incidence of falls during the execution of the study, two models of negative binomial regression will be used where the study groups intervention and control, will be introduced as fixed factors, age and sex as covariables, and the number of days with falls as an exposure measure. The longitudinal analysis of the incidence of falls will be performed through a linear model of mixed effects with the two study groups. In this analysis, the MDS-UPDRS motor subscale scores will be included as a covariate so that it can be adjusted by gravity of the motor symptoms. The cost-effectiveness analysis will be performed using ICER for drops and QALYs detailed above. Secondary analyzes will include (1) comparisons between the two study groups of the different efficacy variables using statistical tests according to normal distribution or not of the variables), (2) falls detection (bivariate correlations between the number of drops provided by the diaries of falls, vs. the number of falls objectified by the sensors; 3) prediction of falls. Raw data provided by sensors shall be analyzed to establish the algorithms that predict the risk of falls with diagnostic validity through ROC curves (sensitivity, specificity, positive predictive value, negative predictive value);

1. analysis of the incidence rate of falls (IR) and rate incidence ratio (IRR), where in a longitudinal way they will establish as multidisciplinary intervention improves the risk of falls vs. the usual clinical practice

We will also analyze the change of status (%) between moderate/high risk to low risk of falls compared to baseline between the two study groups established in the CribC questionnaire (visits 6 months, and 9 months). A level of statistical significance α=0.05 shall be established, adjustments for multiple comparisons shall be made where necessary.
